# Supplementary material for: Clinical epidemiology and high genetic diversity amongst Cryptococcus spp. isolates infecting people living with HIV in Kinshasa, Democratic Republic of Congo
Source: PLoS One. 2022 May 19;17(5):e0267842. doi: 10.1371/journal.pone.0267842 (PMC9119562; doi:10.1371/journal.pone.0267842)
Supplement: S2 File — (DOCX) [file pone.0267842.s002.docx]

**STAIN 1**

BZ-105NGA_1

- Submitted sequence: 5.8S ribosomal RNA gene, partial sequence; internal transcribed spacer 2, complete sequence; and large subunit ribosomal RNA gene, partial sequence.
- GenBank accession number (proposed): ON365502
- Source: *Cutaneotrichosporon curvatum.*
- Organism: *Cutaneotrichosporon curvatum; Eukaryota; Fungi; Dikarya; Basidiomycota; Agaricomycotina; Tremellomycetes; Trichosporonales; Trichosporonaceae; Cutaneotrichosporon.*
- Auteurs: Bive Zono Bive, Rosalie Sacheli, Hippolyte Situakibanza Nani-Tuma, Pius Kabututu Zakayi, Alex Ka, Marcel Mbula Mambimbi, Gaultier Muendele, Raphael Boreux, Nicole Landu, Celestin Nzanzu Mudogo, Pierre-Robert M’Buze, Michel Moutschen, Wieland Meyer, Georges Mvumbi Lelo, Marie-Pierre Hayette.
- Title: Clinical epidemiology and high genetic diversity amongst *Cryptococcus* spp. isolates infecting people living with HIV in Kinshasa, Democratic Republic of Congo
- Journal: PLoS One (2022) in production.

Comments

- Sequencing Technology: Sanger dideoxy sequencing.
- Organism="*Cutaneotrichosporon curvatum*"
- Mol_ type="genomic DNA"
- Isolation_ source="Cerebrospinal fluid people living with HIV patient"
- db_xref="taxon:57679"
- Country="Democratic Republic of the Congo"
- Collection_date="Jun-2019"
- misc_RNA <1 > 287.
- Note="contains 5.8S ribosomal RNA, internal transcribed spacer 2, and large subunit ribosomal RNA"
- Sequence

1 tayttkgacg cacttgcgct ctctggtatt ccggagagca tgcctgtttg agtgtcatga

61 aatctcaacc attagggttt cttaatggct tggatttgga cgtttgccag tcaaatggct

121 cgtcttaaaa gagttagtga atttaacatt tgtcttctgg cgtaataagt ttcgctgggc

181 tgatagtgtg aagtttgctt ctaatcgtcc gcaaggacaa ttcttgaact ctggcctcaa

241 atcaggtagg actacccgct gaacttaagc atatcaataa gcggagg

//

**STRAIN 2**

BZ-23LU_1

- Submitted sequence: 5.8S ribosomal RNA gene, partial sequence; internal transcribed spacer 2, complete sequence; and large subunit ribosomal RNA gene, partial sequence.
- GenBank accession number (proposed): ON365503
- Source: *Cutaneotrichosporon curvatum*
- Organism: *Cutaneotrichosporon curvatum; Eukaryota; Fungi; Dikarya; Basidiomycota; Agaricomycotina; Tremellomycetes; Trichosporonales; Trichosporonaceae; Cutaneotrichosporon*.
- Auteurs: Bive Zono Bive, Rosalie Sacheli, Hippolyte Situakibanza Nani-Tuma, Pius Kabututu Zakayi, Alex Ka, Marcel Mbula Mambimbi, Gaultier Muendele, Raphael Boreux, Nicole Landu, Celestin Nzanzu Mudogo, Pierre-Robert M’Buze, Michel Moutschen, Wieland Meyer, Georges Mvumbi Lelo, Marie-Pierre Hayette.
- Title: Clinical epidemiology and high genetic diversity amongst *Cryptococcus* spp. isolates infecting people living with HIV in Kinshasa, Democratic Republic of Congo
- Journal: PLoS One (2022) in production.

Comments

- Sequencing Technology: Sanger dideoxy sequencing.
- Organism="*Cutaneotrichosporon curvatum*"
- Mol_ type="genomic DNA"
- Isolation_ source="Cerebrospinal fluid people living with HIV patient"
- db_xref="taxon:57679"
- Country="Democratic Republic of the Congo"
- Collection_ date="Jun-2019"
- misc_ RNA <1.> 287.
- Note="contains 5.8S ribosomal RNA, internal transcribed spacer 2, and large subunit ribosomal RNA"
- Sequence

1 cwytttkgaa cgcacttgcg ctctctggta ttccggagag catgcctgtt tgagtgtcat

61 gaaatctcaa ccattagggt ttcttaatgg cttggatttg gacgtttgcc agtcaaatgg

121 ctcgtcttaa aagagttagt gaatttaaca tttgtcttct ggcgtaataa gtttcgctgg

181 gctgatagtg tgaagtttgc ttctaatcgt ccgcaaggac aattcttgaa ctctggcctc

241 aaatcaggta ggactacccg ctgaacttaa gcatatcaat aagcggagg

//

**STRAINS 3**

BZ-33LU_1

- Submitted sequence: 5.8S ribosomal RNA gene, partial sequence; internal transcribed spacer 2, complete sequence; and large subunit ribosomal RNA gene, partial sequence.
- GenBank accession number (proposed): ON365504
- Source: *Cutaneotrichosporon curvatum*
- Organism: *Cutaneotrichosporon curvatum; Eukaryota; Fungi; Dikarya; Basidiomycota; Agaricomycotina; Tremellomycetes; Trichosporonales; Trichosporonaceae; Cutaneotrichosporon*.
- Auteurs: Bive Zono Bive, Rosalie Sacheli, Hippolyte Situakibanza Nani-Tuma, Pius Kabututu Zakayi, Alex Ka, Marcel Mbula Mambimbi, Gaultier Muendele, Raphael Boreux, Nicole Landu, Celestin Nzanzu Mudogo, Pierre-Robert M’Buze, Michel Moutschen, Wieland Meyer, Georges Mvumbi Lelo, Marie-Pierre Hayette.
- Title: Clinical epidemiology and high genetic diversity amongst *Cryptococcus* spp. isolates infecting people living with HIV in Kinshasa, Democratic Republic of Congo
- Journal: PLoS One (2022) in production.

Comments

- Sequencing Technology: Sanger dideoxy sequencing.
- Organism="*Cutaneotrichosporon curvatum*"
- Mol_ type="genomic DNA"
- Isolation_ source="Cerebrospinal fluid people living with HIV patient"
- db_xref="taxon:57679"
- Country="Democratic Republic of the Congo"
- Collection_ date="Jun-2019"
- misc_ RNA <1.> 287.
- Note="contains 5.8S ribosomal RNA, internal transcribed spacer 2, and large subunit ribosomal RNA"
- Sequence

1 tgaatcatcg aatctttgaa cgcaacttgc gctctctggt attccggaga gcatgcctgt

61 ttgagtgtca tgaaatctca accattaggg tttcttaatg gcttggattt ggacgtttgc

121 cagtcaaatg gctcgtctta aaagagttag tgaatttaac atttgtcttc tggcgtaata

181 agtttcgctg ggctgatagt gtgaagtttg cttctaatcg tccgcaagga caattcttga

241 actctggcct caaatcaggt aggactaccc gckaactaag caatccag

//

**STRAINS 4**

BZ-29LU_1

- Submitted sequence: 5.8S ribosomal RNA gene, partial sequence; internal transcribed spacer 2, complete sequence; and large subunit ribosomal RNA gene, partial sequence.
- GenBank accession number (proposed): ON365505
- Source: *Cutaneotrichosporon curvatum*
- Organism: *Cutaneotrichosporon curvatum; Eukaryota; Fungi; Dikarya; Basidiomycota; Agaricomycotina; Tremellomycetes; Trichosporonales; Trichosporonaceae; Cutaneotrichosporon*.
- Auteurs: Bive Zono Bive, Rosalie Sacheli, Hippolyte Situakibanza Nani-Tuma, Pius Kabututu Zakayi, Alex Ka, Marcel Mbula Mambimbi, Gaultier Muendele, Raphael Boreux, Nicole Landu, Celestin Nzanzu Mudogo, Pierre-Robert M’Buze, Michel Moutschen, Wieland Meyer, Georges Mvumbi Lelo, Marie-Pierre Hayette.
- Title: Clinical epidemiology and high genetic diversity amongst *Cryptococcus* spp. isolates infecting people living with HIV in Kinshasa, Democratic Republic of Congo
- Journal: PLoS One (2022) in production.

Comments

- Sequencing Technology: Sanger dideoxy sequencing.
- Organism="*Cutaneotrichosporon curvatum*"
- Mol_ type="genomic DNA"
- Isolation_ source="Cerebrospinal fluid people living with HIV patient"
- db_xref="taxon:57679"
- Country="Democratic Republic of the Congo"
- Collection_ date="Jun-2019"
- misc_ RNA <1.> 287.
- Note="contains 5.8S ribosomal RNA, internal transcribed spacer 2, and large subunit ribosomal RNA"
- Sequence

1 tgaatcatcg aatctttgaa cgcaacttgc gctctctggt attccggaga gcatgcctgt

61 ttgagtgtca tgaaatctca accattaggg tttcttaatg gcttggattt ggacgtttgc

121 cagtcaaatg gctcgtctta aaagagttag tgaatttaac atttgtcttc tggcgtaata

181 agtttcgctg ggctgatagt gtgaagtttg cttctaatcg tccgcaagga caattcttga

241 actctggcct caaatcaggt aggactaccc gckaactaag caatcat

//

**STRAINS 5**

BZ-94NGA_1

- Submitted sequence: 5.8S ribosomal RNA gene, partial sequence; internal transcribed spacer 2, complete sequence; and large subunit ribosomal RNA gene, partial sequence.
- GenBank accession number (proposed): ON365506
- Source: *Cutaneotrichosporon curvatum*
- Organism: *Cutaneotrichosporon curvatum; Eukaryota; Fungi; Dikarya; Basidiomycota; Agaricomycotina; Tremellomycetes; Trichosporonales; Trichosporonaceae; Cutaneotrichosporon*.
- Auteurs: Bive Zono Bive, Rosalie Sacheli, Hippolyte Situakibanza Nani-Tuma, Pius Kabututu Zakayi, Alex Ka, Marcel Mbula Mambimbi, Gaultier Muendele, Raphael Boreux, Nicole Landu, Celestin Nzanzu Mudogo, Pierre-Robert M’Buze, Michel Moutschen, Wieland Meyer, Georges Mvumbi Lelo, Marie-Pierre Hayette.
- Title: Clinical epidemiology and high genetic diversity amongst *Cryptococcus* spp. isolates infecting people living with HIV in Kinshasa, Democratic Republic of Congo
- Journal: PLoS One (2022) in production.

Comments

- Sequencing Technology: Sanger dideoxy sequencing.
- Organism="*Cutaneotrichosporon curvatum*"
- Mol_ type="genomic DNA"
- Isolation_ source="Cerebrospinal fluid people living with HIV patient"
- db_xref="taxon:57679"
- Country="Democratic Republic of the Congo"
- Collection_ date="Jun-2019"
- misc_ RNA <1.> 287.
- Note="contains 5.8S ribosomal RNA, internal transcribed spacer 2, and large subunit ribosomal RNA"
- Sequence

1 taytttkacg cacttgcgct ctctggtatt ccggagagca tgcctgtttg agtgtcatga

61 aatctcaacc attagggttt cttaatggct tggatttgga cgtttgccag tcaaatggct

121 cgtcttaaaa gagttagtga atttaacatt tgtcttctgg cgtaataagt ttcgctgggc

181 tgatagtgtg aagtttgctt ctaatcgtcc gcaaggacaa ttcttgaact ctggcctcaa

241 atcaggtagg actacccgct gaacttaagc atatcaataa gcggagg

//

**STRAINS 6**

BZ-42NGA_1

- Submitted sequence: 5.8S ribosomal RNA gene, partial sequence; internal transcribed spacer 2, complete sequence; and large subunit ribosomal RNA gene, partial sequence.
- GenBank accession number (proposed): ON365509
- Source: *Papiliotrema laurentii*
- Organism: *Papiliotrema laurentii*, *Eukaryota*; *Fungi*; *Dikarya*; *Basidiomycota*; *Agaricomycotina*; *Tremellomycetes*; *Tremellales*; *Rhynchogastremataceae*; *Papiliotrema*.
- Auteurs: Bive Zono Bive, Rosalie Sacheli, Hippolyte Situakibanza Nani-Tuma, Pius Kabututu Zakayi, Alex Ka, Marcel Mbula Mambimbi, Gaultier Muendele, Raphael Boreux, Nicole Landu, Celestin Nzanzu Mudogo, Pierre-Robert M’Buze, Michel Moutschen, Wieland Meyer, Georges Mvumbi Lelo, Marie-Pierre Hayette.
- Title: Clinical epidemiology and high genetic diversity amongst *Cryptococcus* spp. isolates infecting people living with HIV in Kinshasa, Democratic Republic of Congo.
- Journal: PLoS One (2022) in production.

Comments

- Sequencing Technology: Sanger dideoxy sequencing.
- Organism = "*Papiliotrema laurentii*"
- Mol_ type = "genomic DNA"
- Isolation_ source = "Cerebrospinal fluid people living with HIV patient"
- db_ xref = "taxon: 5418"
- Country = "Democratic Republic of the Congo"
- Collection_ date = "Jun-2019"
- misc_ RNA <1.> 318.
- Note="contains 5.8S ribosomal RNA, internal transcribed spacer 2, and large subunit ribosomal RNA"
- Sequence

1 caytttkaac gcaccttgcg ccttttggta ttccgaaagg catgcctgtt tgagtgtcat

61 gaaatctcaa tcccctcggg tttttcgacc tgggtgggac ttggacttgg gcgtctgccg

121 gtaacacggc tcgcctcaaa tgactcagtg gatctctctg catccgtgtc agacgtaata

181 agtttcgtct cgacccttgc ttctgagtct gctcacaacc cgccatcgcg cactttagac

241 tctgacctca aatcaggtag gactacccgc tgaacttaag catatcaata agcggaggag

301 gtcatarctg tttcctga

//
