# Supplementary material for: Clinical epidemiology and high genetic diversity amongst Cryptococcus spp. isolates infecting people living with HIV in Kinshasa, Democratic Republic of Congo
Source: PLoS One. 2022 May 19;17(5):e0267842. doi: 10.1371/journal.pone.0267842 (PMC9119562; doi:10.1371/journal.pone.0267842)

PLOS ONE

Clinical epidemiology and high genetic diversity amongst Cryptococcus spp. isolates infecting people living with HIV in Kinshasa, Democratic Republic of Congo

--Manuscript Draft--

**Manuscript Number:**	PONE-D-21-40474
**Article Type:**	Research Article
**Full Title:**	Clinical epidemiology and high genetic diversity amongst Cryptococcus spp. isolates infecting people living with HIV in Kinshasa, Democratic Republic of Congo
**Short Title:**	Clinical and molecular overview of cryptococcosis in PLHIV in Kinshasa, DRC
**Corresponding Author:**	BIVE BIVE ZONO, M.D<br>University of Kinshasa: Universite de Kinshasa<br>Kinshasa, Kinshasa CONGO, THE DEMOCRATIC REPUBLIC OF THE
**Keywords:**	Cryptococcus spp.; species diversity; ITS sequencing; MALDI-TOF MS; Multilocus sequence typing; people living with HIV; neuromeningeal cryptococcosis; Kinshasa; DRC
**Abstract:**	Neuromeningeal cryptococcosis (NMC) due to Cryptococcus spp. complex is one of the life-threatening opportunistic infections during HIV infection, mainly in sub-Saharan Africa. We focused on the molecular characterization of Cryptococcus isolates from people living with HIV (PLHIV) in Kinshasa (DRC) and investigated possible associations between NMC severity factors and the Cryptococcus neoformans (Cn) multilocus sequence typing (MLST) profiles. The isolates were characterized using PCR serotyping, MALDI-TOF MS, internal transcribed spacer (ITS) sequencing and MLST analysis. NMC severity factors, such as hypoglycorrhachia (<50mg/dL), very raised cerebral spinal fluid opening pressure (>30 cm water), and pejorative outcome in patients were compared with the Cn MLST sequences type (ST). Twenty-three out of 29 Cryptococcus isolates have been identified as serotype A using PCR (79.3%; 95% IC: 65.5-93.1), while six (20.7%; 95% IC: 6.9-34.5) were not serotypable. The 29 isolates have been identified by ITS sequencing as follows: Cryptococcus neoformans (23/29, 79.3%), Cryptococcus curvatus (5/29, 17.2%), and Cryptococcus laurentii (1/29, 3.5%). All Cn isolates were identified as molecular type VNI using the MLST ISHAM scheme, including seven different STs: ST93 (n=15), ST5 (n=2), ST53 (n=1), ST31 (n=1), ST4 (n=1), ST69 (n=1), and one novel ST identified in the present work and subsequently assigned as ST659 (n=2). Among NMC severity factors, only the patient pejorative outcome was associated with infections by less common STs isolates (7/8, 87.5%, p=0.02) (ST53, ST31, ST5, ST4, ST659, and ST69). Molecular analysis of Cryptococcus spp. isolates showed a wide species diversity and genetic heterogenicity of Cn within the VNI molecular type. Furthermore, infections due to less common STs were associated with more pejorative outcomes than those due to ST93.
**Order of Authors:**	BIVE BIVE ZONO, M.D<br>Rosalie SACHELI, PhD<br>Alex KA, PhD<br>Gaultier MUENDELE, M.D<br>Raphaël BOREUX, Medical Biologist<br>Nicole LANDU, M.D<br>Pierre-Robert M'BUZE, M.D<br>Michel MOUTSCHEN, Professor<br>Wieland MEYER, Professor<br>Marie-Pierre HAYETTE, Professor<br>Hippolyte SITUAKIBANZA<br>Marcel MBULA, M.D

Powered by Editorial Manager® and ProduXion Manager® from Aries Systems Corporation

	Pius KABUTUTU, Professor
	Georges MVUMBI, Professor
	Celestin MUDOGO, PhD
**Additional Information:**	
**Question**	**Response**
**Financial Disclosure**<br><br>Enter a financial disclosure statement that describes the sources of funding for the work included in this submission. Review the submission guidelines for detailed requirements. View published research articles from *PLOS ONE* for specific examples.<br><br>This statement is required for submission and **will appear in the published article** if the submission is accepted. Please make sure it is accurate.<br><br>**Unfunded studies**<br>Enter: *The author(s) received no specific funding for this work.*<br><br>**Funded studies**<br>Enter a statement with the following details:<br>• Initials of the authors who received each award<br>• Grant numbers awarded to each author<br>• The full name of each funder<br>• URL of each funder website<br>• Did the sponsors or funders play any role in the study design, data collection and analysis, decision to publish, or preparation of the manuscript?<br>• **NO** - Include this sentence at the end of your statement: *The funders had no role in study design, data collection and analysis, decision to publish, or preparation of the manuscript.*<br>• **YES** - Specify the role(s) played.<br><br>* typeset	The current study has been funded by Académie de Recherche et d'Enseignement Supérieur (ARES-Belgium). The funders had no role in study design, data collection and analysis, decision to publish, or preparation of the manuscript.
**Competing Interests**<br><br>Use the instructions below to enter a competing interest statement for this submission. On behalf of all authors, disclose any competing interests that could be perceived to bias this	The authors have declared that no competing interests exist.

work—acknowledging all financial support and any other relevant financial or non-financial competing interests.<br><br>This statement is required for submission and **will appear in the published article** if the submission is accepted. Please make sure it is accurate and that any funding sources listed in your Funding Information later in the submission form are also declared in your Financial Disclosure statement.<br><br>View published research articles from *PLOS ONE* for specific examples.<br><br>**NO authors have competing interests**<br><br>Enter: *The authors have declared that no competing interests exist.*<br><br>**Authors with competing interests**<br><br>Enter competing interest details beginning with this statement:<br><br>*I have read the journal's policy and the authors of this manuscript have the following competing interests: [insert competing interests here]*<br><br>* typeset	
**Ethics Statement**<br><br>Enter an ethics statement for this submission. This statement is required if the study involved:<br><br>• Human participants<br>• Human specimens or tissue<br>• Vertebrate animals or cephalopods<br>• Vertebrate embryos or tissues<br>• Field research<br><br>Write "N/A" if the submission does not require an ethics statement.	This work was carried out in strict compliance with ethical rules, with the approval of the Ethics Committee of the Public Health School of the Faculty of Medicine of the University of Kinshasa under the approval number ESP/CE/071/2019. All patients included in this study were informed of the risks associated with the study and gave their written informed consent to participate.<br>In case of poor clinical condition or in minor patients, the approval of the guardian was obtained. In addition, all data were analysed anonymously.

Powered by Editorial Manager® and ProduXion Manager® from Aries Systems Corporation

General guidance is provided below. Consult the submission guidelines for detailed instructions. **Make sure that all information entered here is included in the Methods section of the manuscript.**<br><br>**Format for specific study types**<br><br>**Human Subject Research (involving human participants and/or tissue)**<br>• Give the name of the institutional review board or ethics committee that approved the study<br>• Include the approval number and/or a statement indicating approval of this research<br>• Indicate the form of consent obtained (written/oral) or the reason that consent was not obtained (e.g. the data were analyzed anonymously)<br><br>**Animal Research (involving vertebrate animals, embryos or tissues)**<br>• Provide the name of the Institutional Animal Care and Use Committee (IACUC) or other relevant ethics board that reviewed the study protocol, and indicate whether they approved this research or granted a formal waiver of ethical approval<br>• Include an approval number if one was obtained<br>• If the study involved *non-human primates*, add *additional details* about animal welfare and steps taken to ameliorate suffering<br>• If anesthesia, euthanasia, or any kind of animal sacrifice is part of the study, include briefly which substances and/or methods were applied<br><br>**Field Research**<br><br>Include the following details if this study involves the collection of plant, animal, or other materials from a natural setting:<br>• Field permit number<br>• Name of the institution or relevant body that granted permission	
**Data Availability**<br><br>Authors are required to make all data underlying the findings described fully	Yes - all data are fully available without restriction

available, without restriction, and from the time of publication. PLOS allows rare exceptions to address legal and ethical concerns. See the PLOS Data Policy and FAQ for detailed information.<br><br>A Data Availability Statement describing where the data can be found is required at submission. Your answers to this question constitute the Data Availability Statement and **will be published in the article**, if accepted.<br><br>**Important:** Stating 'data available on request from the author' is not sufficient. If your data are only available upon request, select 'No' for the first question and explain your exceptional situation in the text box.<br><br>Do the authors confirm that all data underlying the findings described in their manuscript are fully available without restriction?	
**Describe where the data may be found in full sentences. If you are copying our sample text, replace any instances of XXX with the appropriate details.**<br><br>• If the data are **held or will be held in a public repository**, include URLs, accession numbers or DOIs. If this information will only be available after acceptance, indicate this by ticking the box below. For example: *All XXX files are available from the XXX database (accession number(s) XXX, XXX.*).<br>• If the data are all contained **within the manuscript and/or Supporting Information files**, enter the following: *All relevant data are within the manuscript and its Supporting Information files.*<br>• If neither of these applies but you are able to provide **details of access elsewhere**, with or without limitations, please do so. For example:<br><br>*Data cannot be shared publicly because of [XXX]. Data are available from the XXX Institutional Data Access / Ethics*	All clinical and genomic data will be available in online databases after publication. Before that, they can be transferred without resistance for useful purposes.

Committee (contact via XXX) for researchers who meet the criteria for access to confidential data.<br><br>*The data underlying the results presented in the study are available from (include the name of the third party and contact information or URL).*<br>• This text is appropriate if the data are owned by a third party and authors do not have permission to share the data.<br><br>* typeset	
Additional data availability information:	Tick here if your circumstances are not covered by the questions above and you need the journal's help to make your data available.

Powered by Editorial Manager® and ProduXion Manager® from Aries Systems Corporation

Manuscript
Click here to access/download;Manuscript;Cryptococcus spp. MLST in Kinshasa, RDC.docx

Clinical epidemiology and high genetic diversity amongst *Cryptococcus* spp. isolates infecting people living with HIV in Kinshasa, Democratic Republic of Congo

Bive ZONO BIVE[1, 6], Rosalie SACHELI[2, 6], Hippolyte SITUAKIBANZA NANI-TUMA[3], Pius KABUTUTU ZAKAYI[1], Alex KA[4], Marcel MBULA MAMBIMBI[3], Gaultier MUENDELE[5], Raphaël BOREUX[6], Nicole LANDU[7], Celestin NZANZU MUDOGO[1], Pierre-Robert M'BUZE[8], Michel MOUTSCHEN[9], Wieland MEYER[4, 10], Georges MVUMBI LELO[1], Marie-Pierre HAYETTE[2, 6]

[1]Molecular Biology Service, Department of Basic Sciences, Faculty of Medicine, University of Kinshasa, Kinshasa, the Democratic Republic of Congo;

[2]National Reference Center for Mycosis, University Hospital Center of Liege, Liege, Belgium;

[3]Infectious Diseases Service, Department of Internal Medicine/Department of Tropical Medicine, Faculty of Medicine, University of Kinshasa, Kinshasa, the Democratic Republic of Congo;

[4]Molecular Mycology Research Laboratory, Centre for Infectious Diseases and Microbiology, Faculty of Medicine and Health, Sydney Medical School, Westmead Clinical School, Marie Bashir Institute for Infectious Diseases and Biosecurity, University of Sydney, Westmead Hospital-Research and Education Network, Westmead Institute for Medical Research, Sydney, Australia;

[5]Advanced HIV-Infection Management Unit, Internal Medicine Department, Centre Hospitalier Mère et Enfant de NGABA, Kinshasa, the Democratic Republic of Congo;

[6]Center for Interdisciplinary Research on Medicines, University of Liege, Liege, Belgium;

[7]Advanced HIV-Infection Management Unit, Internal Medicine Department, Centre Médical et Evangélique Révérend LUYINDU, Kinshasa, the Democratic Republic of Congo;

[8]Advanced HIV-Infection Management Unit, Internal Medicine Department, Centre Hospitalier Roi Baudouin 1er, Kinshasa, the Democratic Republic of Congo;

[9]Department of Infectious Diseases and General Internal Medicine, University Hospital Center of Liege, Liege, Belgium;

[10]Curtin Medical School, Curtin University, Perth, Australia.

Corresponding author: Bive ZONO BIVE, Molecular Biology Service, Department of Basic Sciences, Faculty of Medicine, University of Kinshasa, Kinshasa, the Democratic Republic of Congo; Center for Interdisciplinary Research on Medicines, University of Liege, Liege, Belgium.

Tel: (+243) 818682467
E-mail address: bive.zono@unikin.ac.cd; ORCID ID 0000-0002-0084-5068

1. Rosalie SACHELI, PhD: r.sacheli@chuliege.be
2. Hippolyte SITUAKIBANZA NANI-TUMA, MD, Professor: situakibanza.nani@unikin.ac.cd
3. Pius KABUTUTU ZAKAYI, MD, Professor: pius.kabututu@unikin.ac.cd
4. Alex KAN, PhD: alex.kan@sydney.edu.au; ORCID ID 0000-0001-9933-8340
5. Marcel MBULA MAMBIMBI, MD: marcelmbula@gmail.com
6. Gaultier MUENDELE, MD: j.gaultiermuendele@gmail.com
7. Raphaël BOREUX, Medical Biologist: raphael.boreux@chuliege.be
8. Nicole LANDU, MD: nicolelandu220@gmail.com
9. Celestin NZANZU MUDOGO, MD, PhD: celestin.mudogo@unikin.ac.cd
10. Pierre-Robert M'BUZE, MD: docteurmbuze@gmail.com
11. Michel MOUTSCHEN, MD, Professor: mmoutschen@chuliege.be
12. Wieland MEYER, Professor: wieland.meyer@sydney.edu.au; ORCID ID 0000-0003-3691-4077
13. Georges MVUMBI LELO, Professor: georges.mvumbi@unikin.ac.cd
14. Marie-Pierre HAYETTE, Professor: mphayette@chuliege.be

Abstract

Neuromeningeal cryptococcosis (NMC) due to *Cryptococcus* spp. complex is one of the life-threatening opportunistic infections during HIV infection, mainly in sub-Saharan Africa. We focused on the molecular characterization of *Cryptococcus* isolates from people living with HIV (PLHIV) in Kinshasa (DRC) and investigated possible associations between NMC severity factors and the *Cryptococcus neoformans (Cn)* multilocus sequence typing (MLST) profiles. The isolates were characterized using PCR serotyping, MALDI-TOF MS, internal transcribed spacer (ITS) sequencing and MLST analysis. NMC severity factors, such as hypoglycorrhachia (<50mg/dL), very raised cerebral spinal fluid opening pressure (>30 cm water), and pejorative outcome in patients were compared with the *Cn* MLST sequences type (ST). Twenty-three out of 29 *Cryptococcus* isolates have been identified as serotype A using PCR (79.3%; 95% IC: 65.5-93.1), while six (20.7%; 95% IC: 6.9-34.5) were not serotypable. The 29 isolates have been identified by ITS sequencing as follows: *Cryptococcus neoformans* (23/29, 79.3%), *Cryptococcus curvatus* (5/29, 17.2%), and *Cryptococcus laurentii* (1/29, 3.5%). All *Cn* isolates were identified as molecular type VNI using the MLST ISHAM scheme, including seven different STs: ST93 (n=15), ST5 (n=2), ST53 (n=1), ST31 (n=1), ST4 (n=1), ST69 (n=1), and one novel ST identified in the present work and subsequently assigned as ST659 (n=2). Among NMC severity factors, only the patient pejorative outcome was associated with infections by less common STs isolates (7/8, 87.5%, *p*=0.02) (ST53, ST31, ST5, ST4, ST659, and ST69). Molecular analysis of *Cryptococcus* spp. isolates showed a wide species diversity and genetic heterogenicity of *Cn* within the VNI molecular type. Furthermore, infections due to less common STs were associated with more pejorative [illegible]comes than those due to ST93.

Keywords

Cryptococcus spp., species diversity, ITS sequencing, MALDI-TOF MS, Multilocus sequence typing, people living with HIV, neuromeningeal cryptococcosis, Kinshasa, DRC

INTRODUCTION

Among opportunistic infections encountered during HIV/AIDS, neuromeningeal cryptococcosis (NMC) is implied in 15% of deaths and 75% of which occur in sub-Saharan Africa [1]. In this region, the annual mortality from this invasive fungal infection is estimated at 504,000 per year, making it the fourth leading cause of death from infectious diseases [2].

Among 510,000 people living with HIV (PLHIV) in the Democratic Republic of Congo (DRC), only 75% are on antiretroviral treatment (ART) and the NMC prevalence is estimated at 8.8%, with a death rate of approximately one out of three patients [3], [4].

Grounded on epidemiological, pathobiology, geographical distribution, ecological niches, clinical presentation, therapeutic, and genetic differences [5], the *Cryptococcus neoformans/C. gattii* species complex (*Cn/Cg*), the main etiological agents of cryptococcosis, are classified into two species, four varieties, and eight major molecular types [6]. In this manuscript, we applied the new nomenclature of the *Cryptococcus neoformans/C. gattii* species complex as proposed by Ferry Hagen *et al*. [7].

Apart from the *Cn/Cg* species complex, non-*neoformans*/ *gattii Cryptococcus* species which have long been considered saprophytic and non-pathogenic to humans have recently been associated with cryptococcal infections. Of these species, *C. laurentii* and *C. albidus* are identified in 80% of the cases [8]. Some *Cryptococcus* spp., such as *C. gattii* require a more intensive therapeutic approach to management than *C. neoformans*, and others, such as non-*neoformans* and non-*gattii* are known to have primary resistance to fluconazole and 5-flucytosine [9]. Hence, local epidemiological knowledge including circulating *Cryptococcus* spp. molecular types and their susceptibility profiles to usual antifungal agents would facilitate better cryptococcosis surveillance in the general population, and update patients' management based on local data.

As the association of *Cn/Cg* molecular type with the antifungal susceptibility profile has previously been established, it is also opportune to verify the association of molecular types (MT) or even MLST sequence types (ST) with the cryptococcosis clinical presentation. Therefore, we hypothesized that the NMC severity factors could be associated with the isolates ST in the cause of the disease [10].

Therefore, we describe here the NMC clinical epidemiology amongst PLHIV and the molecular characterization of *Cryptococcus* spp. isolates. In addition, the association between NMC severity factors and *Cryptococcus neoformans* MLST ST was statistically analysed.

MATERIALS AND METHODS

Study design, patients and samples

A cross-sectional study was conducted in three Kinshasa public hospitals supported by Doctors without Borders-Belgium (MSF), from 1 February 2019 to 29 February 2020. Thus, 278 patients were included and among them, NMC was diagnosed based on the cryptococcal antigens (CrAg) detection among patients and/or the presence of yeasts cells detected by India ink staining and/or by culture.

Biological analyses

The CrAg detection was carried out in the CSF of each included patient, using the CrAg LFA IMMY test (Immuno-mycologic, Norman, OK, USA). Direct staining with India ink in the CSF was also carried out, and the CSF was cultured on Sabouraud Dextrose Agar-Chloramphenicol medium (SDA-C, bioMérieux, France) at 30°C for 48 to 72h. The qualitative test Pandy was performed for determining proteinorachia as previously described [11].

Identification by MALDI-TOF MS

MALDI-TOF MS (Matrix-Assisted Laser Desorption Ionization Time-Of-Flight Mass Spectrometry, Bruker Daltonics GmbH, Germany) was used for the identification of all fungal strains. From the culture on SDA-C, an extended direct deposit was performed by adding 1µL of 70% formic acid to the sample on a MALDI target plate (MSP 96 BC ground steel target; Bruker Daltonics). Then, 1µL of saturated cyano-4-hydroxycinnamic acid solution (HCCA matrix; Bruker Daltonics) was added. Each microorganism tested was spotted twice on the same MALDI target plate. Measurement was performed with MALDI Flex control V3.4 (Bruker Daltonics) following the settings suggested by the manufacturer using automated collecting spectra. The spectra of each duplicated spot were compared with those in the reference library (BD 8326 or version V 9.0) [12]. The following score was considered for the identification of the fungal species: MS Score ≥ 1.5 and the 3 first results identical and consistent with the appearance of the colonies on agar.

Molecular analysis

DNA extraction

Genomic DNA was extracted from the fresh 24-hour cultures using the NucleoSpin blood quick pure kit (Macherey-Nagel, Düren, Germany). Two preliminary steps were added to the manufacturer's protocol, namely bead-beating and thermal shock. In a 2mL tube containing 0.5mm glass beads (Roche Diagnostics GmbH, Penzberg, Germany), colonies were mixed with 350µL lysis buffer (Promega Corporation, USA). The mixture was vortexed five times due to 6000 vibrations per minute for 40 seconds (bead-beating). Between each pass, the tube was cooled between -20°C and 1°C for 30 seconds in a Nalgene microtube cooler container (Dutscher, France) for a thermal shock.

Serotyping PCR

A classical serotyping PCR designed for *Cn/Cg* species complex was performed according to the protocol described by Ito-Kuwa *et al*.[13].

ITS sequencing

The ITS2 region of the rRNA gene cluster was amplified using the ITS86 forward primer 5′GTGAATCATCGAATCTTTGAA 3′ and ITS4 reverse primer 5′TCCTCCGCTTATTGATATGC 3′ [14]. The amplified products were purified using the kit clean Seq Agencourt (Beckman Coulter Life Science). The sequencing was done on the automate ABI 3500/3500XL (Applied Biosystem, Life Technologies). Bidirectional sequence data were generated after purification using the BigDye terminator sequencing kit (Applied Biosystems, Life Technologies, Belgium). Sequences generated by the software ABI Sequence Scanner V.1.0 (Applied Biosystems, Life Technologies) were then compared to the CBS database by using The BioloMICS database software (https://wi.knaw.nl/page/Pairwise_alignment), which comprises several databases including Genbank. Only results that repeated the same identification at least three times and had a similarity score greater than 95% were considered valid.

Multilocus sequence typing

Multilocus sequence typing (MLST) was performed using the International Society of Human and Animal Mycology (ISHAM) consensus scheme for the *Cn/Cg* species complexes; including six unlinked housekeeping loci (GPD1, LAC1, URA5, SOD1, CAP59, and PLB1) and the non-coding region IGS1 [6]. After DNA extraction, samples were sequenced using Illumina HiSeq as previously described [15], and the raw contigs sequences were paired, removed of duplicate reads, and trimmed using Geneious Prime 64_2021_1 (https://www.geneious.com). Then, the MLST loci were extracted by mapping to the reference sequences of each MLST locus from the online ISHAM MLST fungal database (https://mlst.mycologylab.org/) and each allele type (AT) was assigned using the same online database. The ATs combination defined the sequences type (ST) which in most cases corresponds to the species Molecular type.

Phylogenetic analysis

Phylogenetic analysis of concatenated sequences of the seven MLST loci was performed using MEGA v.6.06 software (http://www.ebi.ac.uk/tools/msa/clustalo). A dendrogram was produced by the Maximum Likelihood method using sequences alignment with the Kimura 2-parameter method. Gaps were treated as a complete deletion. Statistical support for each clade was assessed using bootstrap analysis with 1000 replicates. Apart from *C. neoformans* and *C. gattii* reference strains (WM) included in the analysis, the MLST sequences of the only *C. neoformans* strain (ZS) previously isolated from Congolese infected patient (DRC) was also included.

Statistical analysis

The analysis was carried out using R-cmdr version 2.6-1 (R Foundation for Statistical Computing, Vienna, Austria). Missing data were considered completely random and the available data were analyzed. The continuous variables were summarised as mean ± standard deviation and compared using Student's t-test. The proportions and their respective 95% confidence intervals were calculated for the categorical data. The main outcome variable was the NMC diagnosis. This variable was compared to other variables of the same category using Pearson's chi-square test or Fisher's exact test if the expected values were less than five. Very raised CSF opening pressure (>30cm water), hypoglycorrhachia (<50mg/dL) and patients' pejorative outcome were considered in performing the association analysis between the NMC severity factors and the identified ST-MLST profile. Also, two isolates categories were formed according to the STs-MLST profile: the main ST isolated on the one hand, and the other STs on the other hand. All tests were two-tailed and a $p < 0.05$ was considered statistically significant.

It is noteworthy that comparative data between PLHIV with *Cryptococcus neoformans* versus *Cryptococcus curvatus*/ *C. laurentii* meningitis are presented in another published paper in BMC infectious diseases (https://doi.org/10.1186/s12879-021-06849-3).

Ethical considerations

This work was carried out in strict compliance with ethical rules, with the approval of the Ethics Committee of the Public Health School of the Faculty of Medicine of the University of Kinshasa under the approval number ESP/CE/071/2019. All patients included in this study were informed of the risks associated with the study and gave their informed consent to participate. Anonymity was guaranteed and the data collected was kept and handled by the research team alone.

RESULTS

Among 278 PLHIV included, 66 (23.7%, 95% CI: 18.7 – 28.8) had NMC. However, the NMC prevalence was almost similar in men (24.8%, 95% CI: 14.9 – 34.7, 25/101 included men patients) as in women patients (23.2%, 95% CI: 15.7 – 30.7, 41/177 included women patients).

Patients' characteristics

The demographic and clinical characteristics of the patients are presented in Table 1. The mean age of the included patients was 42.2±12.1 years old. Most of them were female (63.7%), married or cohabitating (49.3%), and had a secondary education level (55·8%). Regarding the patients' clinical stage before NMC diagnosis, NMC tends to develop during HIV-infection stage IV (95.5%, $p = 0.0008$). PLHIV with NMC have four times more probability to present headaches (OR 3.8; IC 95%: 1.7 – 8.8; p=0.0001), three times more probability to present convulsions (OR 2.7; IC 95%: 1.01-7.1, p=0.02); and six times more probability to present visual disturbances than no NMC patients (OR 5.6; IC 95%: 1.04-37.5, p=0.02). The vast majority of NMC patients had clear CSF (93.9%) and a significantly raised CSF

opening pressure (65.4%, p<0.0001). Furthermore, the pejorative outcome was not significantly different between NMC than non-NMC patients (37.5 versus 35.4%, respectively).

Table 1: Demographic and clinical characteristics of patients

Characteristics[1]	Overall data (%)[2]	NMC		p-value	Crude OR (95% CI)
		No (%)	Yes (%)		
Demographic characteristics					
Mean age ± SD (years) (n=278)	42.2 ± 12.1	42.8 ± 12.0	40.2 ± 12.4	0.1	
Female sex (n=278)	177 (63.67)	136 (64.2)	42 (62.1)	0.7	
Marital status (n=278)				0.6	
Single	89 (32.01)	71 (33.5)	18 (27.3)		
Married/cohabitating	137 (49.28)	102 (48.1)	35 (53.0)		
Divorced/widower	52 (18.7)	39 (18.4)	13 (19.7)		
Education level attained (n=278)				0.6	
None/primary	69 (24.82)	52 (24.5)	17 (25.8)		
Secondary	155 (55.76)	121 (57.1)	34 (51.5)		
Higher education/university	54 (19.42)	39 (18.4)	15 (22.7)		
Clinical characteristics					
HIV clinical stage (n=242)				0.0008	-
Stage I	1 (0.41)	1 (0.5)	0 (0.0)		
Stage II	2 (0.83)	2 (0.9)	0 (0.0)		
Stage III	51 (21.07)	48 (22.6)	3 (4.5)		
Stage IV	224 (92.6)	161 (75.9)	63 (95.5)		
Headaches (n=269)	174 (64.68)	120 (58.8)	54 (84.4)	0.0001	3.8 (1.7-8.8)
Fever (°C) (n=269)	179 (66.54)	141 (68.8)	38 (59.4)	0.1	
Weight loss (n=269)	144 (53.53)	107 (52.2)	37 (57.8)	0.4	
Consciousness disorder (n=269)	100 (37.17)	82 (40.0)	18 (28.1)	0.08	
Memory impairment (n=268)	74 (27.61)	59 (28.9)	15 (23.4)	0.3	
Neck stiffness (n=269)	49 (18.22)	33 (15.6)	16 (24.2)	0.1	
Vomiting (n=269)	54 (20.07)	42 (20.5)	12 (18.8)	0.7	
Convulsions (n=269)	23 (8.6)	13 (6.3)	10 (15.6)	0.02	2.7 (1.01-7.1)
Vertigo (n=269)	32 (11.9)	23 (11.2)	9 (14.1)	0.5	
Brudzinski sign (n=269)	15 (5.58)	8 (3.8)	7 (10.6)	0.05	
Physical asthenia (n=269)	30 (11.1)	23 (11.2)	7 (10.9)	0.9	
Kernig sign (n=269)	14 (5.2)	9 (4.2)	5 (7.6)	0.3	
Visual disturbances (n=269)	8 (2.97)	3 (1.5)	5 (7.8)	0.02	5.6 (1.04-37.5)
Functional Impotence (n=269)	17 (6.32)	12 (5.9)	5 (7.8)	0.5	
Clear CSF appearance (n=265)	249 (93.9)	200 (94.3)	62 (93.9)	1	
Very raised CSF opening pressure (cm of water) (n=92)	65 (70.6)	7 (10.6)	17 (65.4)	<0.0001	-
Antiretroviral therapy (ART) (n=278)	204 (73.4)	153 (72.2)	51 (77.3)	0.4	-
Pejorative outcome[3] (n=217)	78 (35.9)	57 (35.4)	21 (37.5)	0.7	-

[1]according to available data
[2]column per cent calculated for each group
[3]Death, status quo, discharge against medical advice, or transfer due to complications

Routine diagnostic analysis of NMC

Out of 66 NMC samples confirmed, 63 (95.5%, 95% CI: 89.4-100) had detectable cryptococcal antigen, only 29 (43.3%, 95% CI: 31.8–56.1) had yeasts present after by India ink staining, and the repeated culture was positive only in 43.3% of the cases (29/66). All three CrAg negative samples were recovered from positive cultures.

MALDI-TOF MS, ITS sequencing, and PCR serotyping characterization

Of the 29 positive cultures, MALDI-TOF MS identified 23 as *C. neoformans* (79.3%), four as *C. curvatus* (13.8%), and two (6.9%) could not be identified. While only 23 isolates were identified as serotype A using serotyping PCR (79.3%), ITS sequencing identified all isolates as *C. neoformans* 79.3% (23/29), *C. curvatus* 17.2% (5/29), and *C. laurentii* 3.5% (1/29). The results of the MALDI-TOF MS, ITS sequencing, and serotyping PCR characterization are summarized in Table 2.

Table 2: MALDI-TOF MS, ITS sequencing, and Multiplex PCR serotyping characterization

Analysis	n=29 (%)
MALDI-TOF MS	
Cryptococcus neoformans	23 (79.3)
Cryptococcus curvatus	4 (13.8)
Not identified	2 (6.9)
ITS sequencing	
Cryptococcus neoformans	23 (79.3)
Cryptococcus curvatus	5 (17.2)
Cryptococcus laurentii	1 (3.5)
Serotyping PCR	
Serotype A	23 (79.3)
No identifiable	6 (20.7)

MLST result

Apart from the six strains identified as *C. curvatus or C. laurentii*, the remaining 23 *C. neoformans* isolates belong to the molecular type VNI. MLST analysis identified seven different STs: ST93 (15 isolates, 65.2%), ST5 (two isolates, 8.6%), ST53 (one isolate, 4.3%), ST31 (one isolate, 4.3%), ST4 (one isolate, 4.3%), ST69 (one isolate, 4.3%), and one novel ST that was not yet reported in the online fungal MLST database and was later assigned as ST659 (two isolates, 8.6%).

Phylogenetic analysis

Phylogenetic analysis using maximum likelihood identified two major clusters among the studied isolates investigated (BZ isolates in figure 1), grouping ST659, ST69, ST4, ST5, and ST93 including 21 isolates out of 23 analysed. The two remaining STs (ST31 and ST53) were slightly less correlated with the other cluster. The single Congolese (DRC) isolate previously characterised and stored in the MLST fungal database was deeply embedded in the first cluster (Fig1).

Fig1. Phylogenetic tree based on concatenated sequences of the seven MLST loci: CAP59, GPD1, IGS1, LAC1, PLB1, SOD1, and URA5 using maximum likelihood. Numbers near the nodes represent the bootstrap values obtained by 1000 repetitions.

NMC severity factors and MLST ST of *Cryptococcus neoformans* isolates

Among NMC severity factors described and considered in the present study, only the pejorative therapeutic outcome was associated with infections due to the less common MLST STs isolates (ST5, ST659, ST53, ST31, ST4, and ST69) versus the main ST (ST93) (87.5% vs. 40%, respectively; p=0.02). Table 3 summarizes the NMC severity factors compared to the MLST ST of the *C. neoformans* isolates.

Table 3: NMC severity factors compared to the MLST STs of the *C. neoformans* isolates.

Variable	*Cryptococcus neoformans* ST identified		*p*
	Main study ST[1] n[3] (%)[4]	Less common STs[2] n[3] (%)[4]	
Glycorhachia (mg/dl) (n=10)			1
Low (≤ 50)	7 (87.5)	2 (100)	
High (≥ 60)	1 (12.5)	0	
Opening pressure (cm of water) (n=8)			0.4
Moderately high (<30)	2 (40)	0	
Very high (≥30)	3 (60)	3 (100)	
Therapeutic outcome (n=23)			0.02
Good[5]	9 (60)	1 (12.5)	
Bad[6]	6 (40)	7 (87.5)	

[1]ST93
[2]STs5, 659, 53, 31, 4, 69.
[3]With available data
[4]Percentage of columns calculated for each group
[5]Recovery and discharge from hospital
[6]Death, status quo, discharge against medical advice, or transfer due to complications

DISCUSSION

We described the clinical epidemiology of PLHIV, the routine analysis and the molecular characterization of the *Cryptococcus* spp. isolates. In addition, the association between the NMC severity factors and *Cryptococcus neoformans* MLST ST was statistically tested.

Neuromeningeal cryptococcosis (NMC) prevalence in people living with HIV (PLHIV) was estimated at 23.7% (95% CI: 18.7 – 28.8). This hospital prevalence is much higher than that reported in France [16], in other Africa states [17], [18], and previously in the DRC [4]. This could be explained by (a) the difference in HIV infection prevalence in various countries and regions, (b) the HIV management and the opportunistic infections prevention policy applied in each country, (c) the HIV patients focused on in each study, and (d) the sensitivity and specificity of biological analysis used for diagnostic confirmation. Reflecting the PLHIV demographic profile in the DRC, female patients aged 42.19 ± 12.13 years old, married/cohabitating and with secondary education level were non-significantly most affected. McClelland E. *et al*. found that virulent *C. neoformans* phenotypes from females had longer doubling times and released more capsular glucuronoxylomannan (GXM) in the 17-β estradiol presence. Plus, macrophages from women phagocytized more *C. neoformans* than those from men. The men hence had a higher fungal load than women and their macrophages were more likely to be destroyed by *C. neoformans* [19]. This protective trend in women was not significantly noted in the current study. Headache, convulsions and visual disturbances were significantly associated with NMC. This data is largely consistent with the literature [20]. Slightly more than the proportion reported in this study (7.7%), visual disturbances are known to be associated with NMC in 18% of cases following raised intracranial pressure [21]. Among the clinical parameters (headache, sensorium depression, papilledema, and raised CSF opening pressure) and the radiological ones (flattening of the posterior sclera, increased CSF in the subarachnoid space around the optic nerve, optic nerve tortuosity and empty parietal saddle) defining these disorders, only headaches and raised CSF opening pressure were found in the present study [21]. NMC was associated with higher raised CSF opening pressure, CD_4 count < 100 cells/mm^3 and HIV-infection stage IV. One of the most critical outcome determinants in PLHIV with NMC is the raised opening CSF pressure which is generally correlated with high CSF fungal load, morbidities and increased risk of death [22]. In agreement with the data described by Bicanic *et al*, 63% of patients in the present study had very high opening CSF pressure [23].

Cryptococcal antigen detection was the main diagnostic tool for NMC in the present study (95% positivity rate out of 66 confirmed samples). Compared to other studies, culture positivity rate, and *Cryptococcus* India ink staining identification were very low in the present study [24]. For Kabanda T. *et al*, CrAg detection has shown more sensitivity in clinical situations (100%) than culture (95.7%) and India ink (93.6%) [25]. The low positivity rate of direct microscopy and culture found in the present study could be caused by the precarious storage conditions of samples before analyses and/or probable low CSF fungal load in certain samples [26]. The ITS sequencing gave a better isolates identification (29/29). Described as the main species responsible for cryptococcosis diseases in PLHIV [27], *C.*

neoformans was identified for 73.3% of all isolates. The remaining cases were identified as *C. curvatus* (17.2%) and *C. laurentii* (3.5%). Initially considered saprophytic and non-pathogenic to humans, the non-*neoformans* and non-*gattii Cryptococcus* species are increasingly found in clinical infections in recent years [8]. Although the *C. neoformans* NMC clinical presentation is more severe in PLHIV than in non-*neoformans*/ non-*gattii* NMC, *C. curvatus* and *C. laurentii* have a high tendency to be fluconazole and 5-flucytosine resistant. In addition, these two latter species are more difficult to identify by routine laboratory methods than *C. neoformans* [8]. Thus, out of six non-*neoformans* and non-*gattii* isolates, only four were correctly identified by MALDI-TOF MS and confirmed by ITS sequencing. The lack of certain species reference spectra in the database provided by the mass spectrometry manufacturer (Bruker: BD 83) for identification, and genome differences between *Cryptococcus* species, could explain these results. Worldwide, serotype A isolates remains the most commonly isolated in environmental and clinical settings [28], a trend also observed in the current study.

The MLST analysis of *C. neoformans* isolates revealed large heterogeneity of STs within the single molecular type found in the present study (VNI), involving seven distinct STs. In line with our results, *Cryptococcus neoformans* ST93 is the most isolated in various countries (China, India, Indonesia, South Africa, Thailand, Brazil, Uganda, and Colombia), both in clinical and environmental settings. It has been associated with high mortality in Uganda [29]. In this study, ST93 was associated with a less pejorative treatment outcome than the less common STs. It hence opens up the debate on the relative virulence of each ST compared to the others. Although most of the STs had already been isolated in the DRC neighbouring countries, namely ST4 in Uganda and Tanzania, ST5, ST31, ST69, and ST93 in Uganda only, one ST (ST53) had previously been isolated only in Thailand and in no other country worldwide (https://mlst.mycologylab.org/). Plus, one isolate had an ST identified for the first time in the present study, subsequently assigned as ST659. The Congolese strain (ZS CN ST32) isolated 30 years earlier is closely related to the isolates of the dominant ST (ST93) identified in the current study.

As described by Trilles *et al.* regarding the low antifungal susceptibility of VGI isolates compared to the other molecular types tested [30], the less common STs isolates identified in the current study were associated with poor therapeutic outcomes.

CONCLUSIONS

A more severe epidemiological profile of NMC than previously reported from the DRC was found using a panel of diagnostic tests in symptomatic PLHIV. Apart from the species diversity identified amongst the yeasts isolated from CSF samples, including *C. neoformans*, *C. curvatus* and *C. laurentii*, the STs within the single molecular type VNI identified in the present study showed great heterogeneity, including seven different STs with one major ST (ST93) and six less common STs (ST5, ST659, ST53, ST31, ST4, and ST69). In addition, the less common STs isolates were associated with the patient

pejorative outcomes. More robust studies including larger sampling numbers and antifungal susceptibility of isolates could improve the understanding of the data from this study.

AUTHOR CONTRIBUTIONS

Conceptualization: BZB.

Clinical and biological analysis: BZB, RS, RB and AK.

Samples and patient clinical data collection: GM, NL and PRM.

Data curation: BZB.

Funding acquisition: GML and MPH.

Investigation: BZB.

Methodology: BZB.

Project administration: MPH.

Resources: BZB and RS.

Supervision: MPH.

Validation: BZB, GM, NL, PRM, RS, RB, AK, HSN, PKZ, MMM, CNM, WM, GML and MPH.

Visualization: BZB.

Writing – original draft: BZB.

Writing – review & editing: HSN, PKZ, MMM, CNM, MM, WM, GML, and MPH.

TRANSPARENCY DECLARATION

The authors declare no conflict of interest either in the conduct of the study or in the publication of this report.

FUNDING

Académie de Recherche et d'Enseignement Supérieur (ARES-Belgium). The study sponsor had no role in study design, data collection, data analysis, data interpretation, or writing of the report.

REFERENCES

1. Rajasingham R, Smith RM, Park BJ, Jarvis JN, Govender NP, Chiller TM, et al. Global burden of disease of HIV-associated cryptococcal meningitis: an updated analysis. Lancet Infect Dis. 2017;17(8):873–881.
2. Park BJ, Wannemuehler KA, Marston BJ, Govender N, Pappas PG, Chiller TM. Estimation of the current global burden of cryptococcal meningitis among persons living with HIV/AIDS. Aids. 2009;23(4):525–530.
3. UNAIDS and AIDSinfo. Country factsheets DEMOCRATIC REPUBLIC OF CONGO 2020 HIV and AIDS Estimates Adults and children living with Country factsheets DRC | 2020 HIV testing and treatment cascade People living with HIV Coverage of adults and children. Unaids. 2021;1–6.
4. Zono B, Kamangu E, Situakibanza H, Amaela E, Bepouka B, Mbula M, et al. Epidemiological, clinical and biological profile of neuromeningeal cryptococcosis among people living with HIV in Kinshasa, Democratic Republic of Congo. Pan Afr Med J. 2020;37(302):1–9.
5. Chen SCA, Korman TM, Slavin MA, Marriott D, Byth K, Bak N, et al. Antifungal therapy and management of complications of cryptococcosis due to Cryptococcus gattii. Clin Infect Dis. 2013;57(4):543–551.
6. Meyer W, Aanensen DM, Boekhout T, Cogliati M, Diaz MR, Esposto MC, et al. Consensus multi-locus sequence typing scheme for Cryptococcus neoformans and Cryptococcus gattii Maria. Med Mycol. 2009;47(6):561–570.
7. Hagen F, Khayhan K, Theelen B, Kolecka A, Polacheck I, Sionov E, et al. Recognition of seven species in the Cryptococcus gattii / Cryptococcus neoformans species complex. Fungal Genet Biol. 2015;78:16–48.
8. Smith N, Sehring M, Chambers J, Patel P. Perspectives on non- neoformans cryptococcal opportunistic infections . J Community Hosp Intern Med Perspect. 2017;7(4):214–217.
9. Johnson LB, Bradley SF, Kauffman CA. Fungaemia due to Cryptococcus laurentii and a review of non-neoformans cryptococcaemia. Mycoses. 1998;41(7–8):277–280.
10. Chen C, Sy H, Lin L, Yen H, Wang S, Chen W, et al. Epidemiological characterization and prognostic factors in patients with confirmed cerebral cryptococcosis in central Taiwan. J Venom Anim Toxins Incl Trop Dis. 2015;21(12):10–13.

11. Jacobs RM, Cochrane SM, Lumsden JH, Norris AM. Relationship of cerebrospinal fluid protein concentration determined by dye-binding and urinary dipstick methodologies. Can Vet J. 1990;31(August):587–588.

12. Library MBTIVD, Library MBTIVD, Ivd MBT, Extension L, Library MBTIVD. Notes de publication. (mars 2019):1–61.

13. Ito-Kuwa S, Nakamura K, Aoki S, Vidotto V. Serotype identification of Cryptococcus neoformans by multiplex PCR. Mycoses. 2007;50(4):277–281.

14. Ferrer C, Colom F, Frasés S, Mulet E, Abad JL, Alió JL. Detection and identification of fungal pathogens by PCR and by ITS2 and 5.8S ribosomal DNA typing in ocular infections. J Clin Microbiol. 2001;39(8):2873–2879.

15. Illumina. Nextera XT DNA Library Prep Kit. 2017.

16. Gangneux JP, Bougnoux ME, Hennequin C, Godet C, Chandenier J, Denning DW, et al. Estimation du poids épidémiologique des infections fongiques graves en France. J Mycol Med. 2016;26(4):385–390.

17. Chadli S, Aghrouch M, Taqarort N, Malmoussi M, Ouagari Z, Moustaoui F, et al. Neuromeningeal cryptococcosis in patients infected with HIV at Agadir regional hospital, (Souss-Massa, Morocco). J Mycol Med. 2018;28(1):161–166.

18. Gbangba-Ngai E, Fikouma V, Mossoro-Kpinde CD, Tekpa G, Ouavene JO, Yangba Mongba DSA, et al. La cryptococcose neuroméningée au cours de l’infection à VIH à Bangui, à l’ère du traitement antirétroviral. Bull la Soc Pathol Exot. 2014;107(2):106–109.

19. McClelland EE, Hobbs LM, Rivera J, Casadevall A, Potts WK, Smith JM, et al. The Role of Host Gender in the Pathogenesis of Cryptococcus neoformans Infections. PLoS One. 2013;8(5):1–7.

20. K. ME, R. PJ. Cryptococcosis. Intraocular Inflamm. 2016;30(1):179–206.

21. Kuriakose CK, Mishra AK, Vanjare HA, Raju A, Abraham OC. Visual disturbance in patients with cryptococcal meningitis: The road ahead. J Neurosci Rural Pract. 2017;8(1):151–152.

22. Graybill JR, Sobel J, Saag M, Van Der Horst C, Powderly W, Cloud G, et al. Diagnosis and management of increased intracranial pressure in patients with AIDS and cryptococcal meningitis. Clin Infect Dis. 2000;30(1):47–54.

23. Bicanic T, Brouwer AE, Meintjes G, Rebe K, Limmathurotsakur D, Chierakul W, et al. Relationship of cerebrospinal fluid pressure, fungal burden and outcome in patients with cryptococcal meningitis undergoing serial lumbar punctures. Aids. 2009;23(6):701–706.

24. Ngouana K, Dongtsa J, Kouanfack C. Cryptoccocal meningitis in Yaoundé (Cameroon) HIV infected patients: diagnosis, frequency and Cryptococcus neoformans isolates susceptibility study to fluconazole. J Mycol médicale. 2015;25:11–16.

25. Kabanda T, Siedner MJ, Klausner JD, Muzoora C, Boulware DR. Point-of-care diagnosis and prognostication of cryptococcal meningitis with the cryptococcal antigen lateral flow assay on cerebrospinal fluid. Clin Infect Dis. 2014;58(1):113–116.

26. Saha DC, Xess I, Biswas A, Bhowmik DM, Padma M V. Detection of Cryptococcus by conventional, serological and molecular methods. J Med Microbiol. 2009;58(8):1098–1105.

27. Kwon-chung KJ, Fraser JA, Doering TL, Wang ZA, Janbon G, Idnurm A, et al. gattii , the Etiologic Agents of Cryptococcosis. Cold Spring Harb Prespectives Med. 2014;4:a019760.

28. Bougnoux ME, Angebault C, Leto J, Beretti JL. Identification des levures par spectrométrie de masse de type MALDI-TOF. Rev Francoph des Lab. 2013;2013(450):63–69.

29. Vélez N, Escandón P. Multilocus sequence typing (MLST) of clinical and environmental isolates of cryptococcus neoformans and cryptococcus gattii in six departments of Colombia reveals high genetic diversity. Rev Soc Bras Med Trop. 2020;53:1–8.

30. Trilles L, Meyer W, Wanke B, Guarro J, Lazéra M. Correlation of antifungal susceptibility and molecular type within the Cryptococcus neoformans/C. gattii species complex. Med Mycol. 2012;50(3):328–332.

 Click here to access/download;Figure;Figure 1.docx

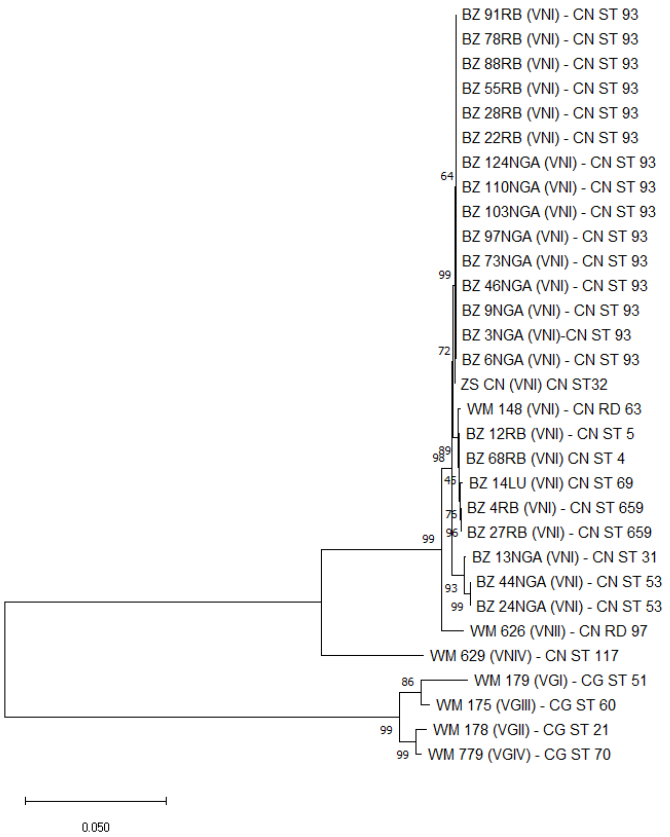

Supplement: Attachment — Submitted filename: PONE-D-21-40474.pdf [file pone.0267842.s005.pdf]
