# Supplementary material for: Clinical epidemiology and high genetic diversity amongst Cryptococcus spp. isolates infecting people living with HIV in Kinshasa, Democratic Republic of Congo
Source: PLoS One. 2022 May 19;17(5):e0267842. doi: 10.1371/journal.pone.0267842 (PMC9119562; doi:10.1371/journal.pone.0267842)
Supplement: Attachment — Submitted filename: Response to Reviewers.docx [file pone.0267842.s006.docx]

Bive ZONO, MD

Academic Assistant and PhD Student

Molecular Biology Service, Basic Sciences Department/Infectious Diseases Service

University of Kinshasa

Universités n°1, Lemba, Kinshasa, Democratic Republic of Congo

Tel: (+243) 818682467

Email: [bive.zono@unikin.ac.cd](mailto:bive.zono@unikin.ac.cd) ; [bive.zono@doct.uliege.be](mailto:bive.zono@doct.uliege.be)

Object: Response to the academic editor and reviewer’s comments

To the editor in charge of the referenced manuscript: PONE-D-21-40474

Titled: “Clinical epidemiology and high genetic diversity amongst *Cryptococcus* spp. isolates infecting people living with HIV in Kinshasa, Democratic Republic of Congo”

Dear Editor,

First of all, I would like to thank you and the reviewers of this manuscript through you, who have clearly and significantly contributed to its improvement.

As mentioned in the manuscript, this work is from the cross-sectional study conducted by our team in tertiary hospitals in Kinshasa (DRC). The initial results led to the elaboration of a comparative study of the baseline clinical and biological profiles of patients with *Cryptococcus neoformans* versus non-*neoforman/non-gattii* (non-*Cryptococcus*) meningitis, exclusively the 29 patients who had a positive culture. This article has been peer-reviewed and published online: <https://doi.org/10.1186/s12879-021-06849-3>.

In the current manuscript, we discuss the general results of this epidemiological investigation (cross-sectional study), based on the clinical profile of the included subjects, routine biological data, molecular identification (PCR serotyping, MALDI TOF MS, ITS sequencing, MLST characterization) and antifungal susceptibility of *Cryptococcus* spp. isolates. All patients’ data are included here. These are therefore not the same data profiles.

In the following lines, you will find our detailed answers.

**Point-by-point response**

I. **Academic editor’ comments**

- Abstract: Could do with a line on implications for programs.

R/ Lines on the implications for programs have been added in the introductory part of the manuscript. Lines 129-133

- Introduction: Given the size of DRC, could do with some further data that show the variability by geography

R/ The additional data on geographical variability in the DRC has been added in the text. Lines 93-97

- Results: Would have been important to include some cascade data that starts with all who were hospitalized, how many had suspected cryptococcal disease and how many had a lumbar puncture.

R/ Following the suggestion, we specified here that all included patients (278) were hospitalized and had a lumbar puncture for investigation. Lines 246-250

- A couple of grammatical areas to look at. Line 189 and 190: “men patients” and “women patients” seems odd. Would rather use, “men” and “women”.

R/ Changes made in the manuscript. Lines 248-250

- Line 195- “stage before NMC diagnosis, NMC tends to develop during HIV-infection stage IV (95.5%, p = 0.0008)”. Would use “tended” instead of “tends”.

R/ Changes made in the text. Line 255

- Line 196: Use four times “higher” probability.

R/ Changes made in the manuscript. Lines 256-259

- In general some sentences are written in present tense while others are presented in past tense.

R/ Uniformity has been revised in the text.

Discussion:

- Line 263: “We described the clinical epidemiology of PLHIV” could be re-written to specify that the authors described the clinical epidemiology of PLHIV with advanced HIV admitted to a tertiary facility and not all PLHIV.

R/ Changes made in the text. Lines 329-331

II. **Reviewer 1’ comments**

- What I see missing are implication of this work programmatically.

R/ The programmatic implications of this work have been incorporated into the manuscript. Lines 129-133

III. **Reviewer 2’ comments**
     3.1. Main comment

- The English language could be proofread.

R/ Based on the suggestion, the manuscript was reviewed and edited by a native English-speaking.

- An evaluation of the susceptibility of the tested strains is also missing.

R/ The susceptibility of the strains to antifungal agents was tested and included in the manuscript.

3.2. General remarks
- The new nomenclature of *Cryptococcus laurentii* and *Cryptococcus curvatus* (i.e. Cutaneotrichospora and Papiliotrema) should be used (Liu 2015, Sujita 2017).

R/ The new nomenclature has been updated in the manuscript and the corresponding references were also updated.

- The epidemiological data used (Park 2009) are obsolete. Those published by Rajasingham in 2017 (ref. 1) should be cited.

R/ Changes made in the manuscript. Lines 88-92

- p 6. The 194 correct 55.8

R/ Changes made in the text. Line 254

- Tables should be reformatted according to journal standards

R/ improvements have been made in the concerned files

- Regarding Table 2, the MALDI-TOF scores should be specified to know the reliability of the identifications. This could explain the wrong identification of Papiliotrema.

R/ A supplementary table containing details of the MALDI-TOF MS findings including scores has been added.

- Figure 1 is not appropriate. It is necessary to generate a Minimum Spanning Tree and to place the STs in a global environment allowing to determine CCs

R/ Minimum Spanning Trees using the geoBURST algorithm were generated and the corresponding figure has been added.

- There is a serious lack of data on the susceptibility of strains to major antifungal drugs. This would strengthen this study and strongly support the Discussion.

R/ The susceptibility of strains to major antifungal drugs was tested and discussed in the manuscript.

- In the Discussion section (p10. L 266-268): the references are too old (ref. 18) and the choice of countries is not relevant. There are very good studies on cryptococcosis in Africa and they deserve to be added to your Discussion. For example, there are studies in Cameroon, Côte d'Ivoire or Kenya (Loyse 2019, Kassi 2019, Gitonga 2019).

R/ Update done in the manuscript. For this discussion part, the first intention was to compare the hospital prevalence of NMC in HIV populations in different regions. Articles with this baseline data have been completed, including the great suggested articles that have been extensively used in the rest of the discussion. Lines 334-337

- P10. L 268-272: ST can also explain your hypothesis.

R/ Suggestion taken into account in the manuscript. Lines 338-342

- P11. L 300-302: *C. laurentii* has long been known to cause infections in humans (Kamalam 1976, Lynch 1981).

R/ Correction taken into account in the manuscript. Lines 374-378

- P 11. L 313-314: Here again, it is imperative to compare the data with those published in other African countries (e.g. ST 5 and 93 are present in Ivory Coast).

R/ References from studies conducted in African regions on MLST characterisation of *Cryptococcus neoformans/Cryptococcus gattii* strains were sought and considered. Lines 393-407

- P 11. L317-322: please cite references

R/ It should be noted here that all STs mentioned in this section were obtained from the online database: international fungal MLST database: <https://mlst.mycologylab.org/>. Details have been provided in the manuscript and corresponding references have been cited.

Done at Kinshasa, 7 February 2022

Bive ZONO, MD
