# Supplementary material for: Clinical epidemiology and high genetic diversity amongst Cryptococcus spp. isolates infecting people living with HIV in Kinshasa, Democratic Republic of Congo
Source: PLoS One. 2022 May 19;17(5):e0267842. doi: 10.1371/journal.pone.0267842 (PMC9119562; doi:10.1371/journal.pone.0267842)
Supplement: Attachment — Submitted filename: PONE-D-2140474 Reviewer comments.docx [file pone.0267842.s004.docx]

**Title**: Clinical epidemiology and high genetic diversity amongst Cryptococcus spp. isolates infecting people living with HIV in Kinshasa, Democratic Republic of Congo

**Overview and general comments**: This article addresses a microorganism of great importance for PLHIV given the fact that many people still present late and with advanced HIV and even a non-significant proportion who disengage from treatment still present with advanced HIV and are at risk of high mortality and morbidity from cryptococcal meningitis. In this light, this is an important paper that could help improve outcomes for people with AHD.

The paper is well written but also very technical. My review and comments are specific for the implications for improving outcomes for PLHIV. On this basis, challenges with conducting lumbar punctures and doing the relevant CSF analyses in resource limited settings make it challenging to understand how these finding might be generalized or used outside tertiary centers. Despite this limitation, the paper is quite useful. Below are a couple of specific comments.

**Specific comments**:

**Abstract**: Very well written. Could do with a line on implications for programs.

**Introduction**: Nicely lays the context, burden of cryptococcal disease. Relevant local data are cited on prevalence are cited. Given the size of DRC, could do with some further data that show the variability by geography

**Method**: Well written and hopefully has been reviewed by a microbiologist.

**Results**: Would have been important to include some cascade data that starts with all who were hospitalized, how many had suspected cryptococcal disease and how many had a lumber puncture. A couple of grammatical areas to look at. Line 189 and 190: “men patients” and “women patients” seems odd. Would rather use , “men” and “women”. Line 195- “stage before NMC diagnosis, NMC tends to develop during HIV-infection stage IV (95.5%, p = 0.0008)”. Would use “tended” instead of “tends”. Line 196: Use four times “higher” probability.

In general some sentences are written in present tense while others are presented in past tense.

Tables are well presented

**Discussion**: The results are well discussed. Line 263: “We described the clinical epidemiology of PLHIV” could be re-written to specify that that the authors described the clinical epidemiology of PLHIV with advanced HIV admitted to a tertiary facility and not all PLHIV. ,

**Conclusion**: well written
